# Characterization of Iron Oxide Nanoparticles Inside the *Myxococcus xanthus* Encapsulin

**DOI:** 10.3390/nano15231793

**Published:** 2025-11-28

**Authors:** Harry B. McDowell, Egbert Hoiczyk, Thomas Walther

**Affiliations:** 1 School of Electrical and Electronic Engineering, University of Sheffield, Mappin Building, Mappin Street, Sheffield S1 3JD, UK; hmcdowell636@gmail.com; 2 School of Biosciences, Krebs Institute, University of Sheffield, Firth Court, Western Bank, Sheffield S10 2TN, UK; egbert.hoiczyk@gmail.com

**Keywords:** bacterial encapsulins, nanocompartments, *M. xanthus*, iron oxide, electron microscopy, goethite

## Abstract

Encapsulins are microbial protein nanocompartments that spatially organize and sequester specific biochemical processes, including iron storage. While their protein shells have been extensively characterized, the composition and structure of their mineral cores remain less understood. Here, we use bright field transmission electron microscopy (BF TEM), high-angle annular dark-field scanning TEM (HAADF STEM), energy-dispersive X-ray (EDX), and electron energy-loss spectroscopy (EELS) in STEM to characterize the iron-containing mineral granules within the *Myxococcus xanthus* encapsulin system at near atomic resolution. We find that the internal nanoparticles are smaller (~2 nm) and more numerous (up to ~2200 per encapsulin) than previously reported. These nanoparticles are typically amorphous and have a composition consistent with FePO_4_ (measured Fe:P ratio of ≈1:1.2). Each encapsulin contains on average ~8500 iron atoms, corresponding to a volumetric density of 2.1 atoms/nm^3^. Phosphorus incorporation inhibits crystallization, whereas growth in phosphorus-free media leads to the formation of nano-crystalline goethite [α-FeO(OH)].

## 1. Introduction

Encapsulins are microbial protein nanocompartments that have been bioinformatically identified in at least 31 of 35 bacterial phyla and 4 of 5 archaeal phyla, making them the most diverse and abundant class of microbial protein-only organelles described to date [1,2]. Structurally, encapsulins self-assemble from multiple copies of a single protomer. These protomers form pentameric and hexameric capsomers, which organize into virus-like icosahedral shells ranging from 24–42 nm in diameter, depending on the number of incorporated subunits [3]. The smallest shells (24 nm) consist of 60 protomers arranged into 12 pentamers [4], whereas larger shells are formed through the addition of variable numbers of hexamers. Assemblies of 180 protomers form shells of approximately 32 nm [5], while 240 protomers constitute the largest encapsulins currently known, measuring 42 nm in diameter [6].

During shell assembly, diverse cargo proteins are selectively encapsulated via either a conserved C-terminal targeting peptide [4,7] or a disordered N-terminal targeting domain [8]. Based on the predicted enzymatic and metabolic activities of these cargo proteins, encapsulin systems have been proposed to fulfil a wide range of physiological roles, including iron storage and homeostasis [4], oxidative stress resistance [5,6], sulphur metabolism and elemental sulphur storage [8], anaerobic ammonium oxidation [9,10], lignin degradation [11,12], and secondary or natural product metabolism [12]. Most of these functional predictions are derived from bioinformatic analyses, and only a subset has been experimentally validated, particularly those associated with redox processes, such as oxidative stress response and iron storage [5,6].

One of the best such characterised encapsulin systems involved in the oxidative stress response is found in *Myxococcus xanthus*. Under oxidative stress, this system sequesters free ferrous iron, protecting the cell from reactive oxygen species generated via Fenton chemistry [5,13]. The encapsulin shell assembles into a 32-nm structure and packages three cargo proteins: two ferritin-like proteins, EncB and EncC, and a ferric reductase, EncD [5,13,14]. Atomic structures of EncB and EncC reveal ferroxidase centers capable of catalyzing the oxidation of Fe^2+^ to Fe^3+^ [13]. Despite substantial progress in elucidating the structures and biochemical functions of the protein components, comparatively little is known about the composition, redox state, and crystallinity of the iron-containing nanoparticles within the encapsulin.

Importantly, intracellular iron mineralization and storage in microbes are not unique to encapsulins but also occur in several other bacterial systems [15,16]. The type of iron mineral formed reflects the biological function of each system. For example, the universally distributed ferritin system, which is also present in *M. xanthus*, encapsulates ferrihydrite (Fe_2_O_3_·0.5H_2_O), a hydrous ferric oxyhydroxide mineral, within its protein shell. Ferrihydrite is amorphous, contains variable amounts of water, and allows relatively easy access to stored iron, which can be mobilized through proteolytic degradation of the protein shell. In contrast, magnetosomes, membrane-bound organelles in magnetotactic bacteria, biomineralize the ferromagnetic iron oxide magnetite (Fe_3_O_4_), enabling cells to align with the Earth’s magnetic field and locate optimal oxygen concentrations in aquatic environments [17,18]. Because magnetosomes serve long-term navigational functions and require stable ferrimagnetic properties, the iron mineral is deposited as crystalline magnetite and does not function as a physiologically accessible iron reservoir. In both ferritin and magnetosome systems, biomineralization depends on proteins involved in iron transport, nucleation, and growth (e.g., ferritin, or magnetosome-associated proteins such as MamP, MamD, and MamN), as well as environmental factors including pH, iron availability, and the presence of additional ions, such as phosphate. Emerging evidence suggests that similar biochemical and cellular parameters govern iron mineralization within encapsulin systems.

Although the material encapsulated within the *Quasibacillus thermotolerans* encapsulin has been structurally and chemically well-characterized [6,19], comparable analyses of *M. xanthus* encapsulin granules remain limited. Previous studies were constrained either by low spatial resolution [5] or by a focus on engineered materials with magnetic properties for biotechnological applications [20,21], leaving the native chemical composition and crystallinity of these iron-containing granules poorly understood. Here, we employ a combination of transmission electron microscopy (TEM) techniques, including bright-field TEM and high-angle annular dark-field (HAADF) scanning TEM (STEM), along with complementary spectroscopic methods, such as energy-dispersive X-ray spectroscopy (EDXS) and electron energy-loss spectroscopy (EELS), to characterize the iron-containing mineral granules within the *M. xanthus* encapsulin. Our findings present the first detailed structural and compositional analysis of individual nanoparticles within native encapsulins isolated from starved *M. xanthus* cells. This comprehensive characterization provides critical insights into encapsulin formation and iron storage mechanisms, and elucidates how nanoparticle structure influences the bacterial capacity to sequester or mobilize iron in response to environmental challenges. These findings advance our understanding of bacterial iron homeostasis and adaptive responses to environmental stress.

## 2. Materials and Methods

### 2.1. Strains and Culturing Conditions

A yellow-pigmented wild-type *M. xanthus* DK1622 strain was used for all experiments [22]. Cells were grown in either casitone-tris (CTT) medium (1% casitone, 10 mM Tris-HCl pH 8.0, 8 mM MgSO_4_, and 1 mM KH_2_PO_4_; final pH 7.6) or tris-phosphate-magnesium (TPM) starvation medium (10 mM Tris-HCl pH 8.0, 8 mM MgSO_4_, and 1 mM KH_2_PO_4_; final pH 7.6), with shaking at 200 revolutions per minute (rpm) and 32 °C [23]. Cells were maintained on solid CTT medium containing 1.5% agar at 32 °C.

*E. coli* Rosetta DE3 was grown in lysogeny broth (LB) (Sigma; 10 g/L tryptone, 5 g/L NaCl, 5 g/L yeast extract) on a shaking platform at 200 rpm, or on LB 1.5% agar plates, supplemented with appropriate concentrations of antibiotics (ampicillin 10 µg/mL, chloramphenicol 25 µg/mL, kanamycin 50 µg/mL) at 37 °C. For protein over-expression, filter-sterilised IPTG was added to the liquid growth media at a final concentration of 0.05 mM.

### 2.2. Isolation of Native Encapsulin Nanocompartments

Encapsulins were isolated using a previously published protocol [5] with several modifications. Briefly, yellow-pigmented *M. xanthus* wild-type cells were grown to an optical density at 600 nm wavelength (OD_600_) of 0.4, transferred to TPM medium and incubated for 18 h to induce encapsulin formation. Cells were harvested, re-suspended in double distilled water (ddH_2_O), chilled on ice for 1 h and lysed by ultrasonication. Cellular debris was removed by centrifugation at 10,000 rpm for 10 min (SLA3000 rotor, Sorvall, now part of Thermo Fisher Scientific, Waltham, MA 02451, USA). Ammonium sulfate [(NH_4_)_2_SO_4_] was added slowly to a final concentration of 15% (*w*/*v*), followed by another centrifugation step (10,000 rpm, 10 min, SLA3000 rotor) to remove precipitates. Polyethylene glycol 8K (2%) was then added to the solution and centrifuged at 20,000 rpm (JA25.50 rotor, John Hancock Industries, Inc., Campbellsville, KY 42718, USA). The pellet was resuspended in ddH_2_O supplemented with 0.5% N-dodecyl-*β*-D-maltoside and 0.3 g/mL of CsCl, layered onto a 2 mL 5.7 M CsCl cushion, and ultracentrifuged at 38,000 rpm for 18 h (SW41 rotor, Beckham Coulter, Indianapolis, IN 46268, USA). Encapsulins were recovered as a brown pellet at the bottom of the ultracentrifuge tube. The CsCl gradient was discarded, and the pellet was dissolved in ddH_2_O and dialysed against ddH_2_O for 24 h. The dialysed sample was layered onto a 10–40% sucrose gradient and centrifuged at 20,000 rpm for 2 h (SW41 rotor). The pellet was discarded and the remaining supernatant centrifuged at 55,000 rpm for 1.5 h (SW55 rotor, Beckham Coulter, Indianapolis, IN 46268, USA). The resulting encapsulin pellet was dissolved in ddH_2_O and stored at 4 °C. Sodium azide was added at a final concentration of 1 mM to prevent bacterial contamination.

### 2.3. Molecular Biology and Cloning

The genes *encA* (*MXAN*_3556) and *encC* (*MXAN*_4464) were amplified from *M. xanthus* genomic DNA. The gene for *encB* (*MXAN*_3557) was codon-optimized for expression in *E. coli* and synthesised (Twist Bioscience). A C-terminal 6xHis tag, preceded by a GGSGGS linker, was appended to *encA*. The tagged *encA* gene was inserted into MCS2 of the petDUET-1 plasmid using Ndel and Xho1 restriction sites. *EncB* was subsequently cloned into MSC1 of the same plasmid using NcoI and HindIII restriction sites. The *encC* gene was cloned into MCS1 of the pCOLADUET-1 plasmid using NcoI and HindIII, allowing expression of encapsulins containing multiple cargo proteins. Ligation was performed with T4 DNA ligase, and constructs were transformed into *E. coli* Rosetta DE3 (Novagen, now part of EMD Biosciences Inc., Madison, WI 53711, USA) for protein expression. All enzymes were obtained from New England Biolabs.

### 2.4. Protein Expression and Purification

*E. coli* Rosetta DE3 cells were transformed via heat shock at 42 °C. A starter culture containing the appropriate selection antibiotics was grown overnight at 37 °C and subsequently used to inoculate a larger culture at a dilution of 1:100. Cells were grown at 37 °C until reaching an OD_600_ of 0.4–0.6, at which point expression was induced with 0.05 mM IPTG. Induced cultures were grown for 18 h at 24 °C with shaking at 200 rpm to allow for protein expression. After 18 h, cells were chilled on ice and harvested by centrifugation at 5000× *g* for 10 min at 4 °C. Cell pellets were re-suspended in a lysis buffer (50 mM Tris, 500 mM NaCl, pH 8.0) and broken by ultrasonication. The lysate was clarified by centrifuging at 8000× *g* for 10 min. His-tagged encapsulins were purified using a Ni-NTA affinity chromatography column (Qiagen, Venlo, The Netherlands) pre-equilibrated with buffer containing 50 mM Tris, 500 mM NaCl, and 10 mM imidazole (pH 8.0). The column was washed three times with the buffer containing 20 mM imidazole to remove non-specifically bound proteins. The tagged encapsulins were eluted from the column using the buffer containing 250 mM imidazole in three successive elution steps to ensure complete recovery. Presence of the protein in the elution fraction was confirmed by SDS-PAGE analysis.

### 2.5. Loading Encapsulins with Iron

Encapsulins were alternatively loaded with iron under phosphorus-free conditions. Initially, empty encapsulins were purified from *E. coli* Rosetta (DE3), and the absence of iron mineralization was confirmed by TEM. A 20 mM stock solution of Fe(NH_4_)_2_(SO_4_)_2_ was prepared in 0.1% (*v*/*v*) HCl. This stock solution was then diluted tenfold into a 30 μM protein solution. The mixture was agitated and incubated under aerobic conditions for 1 h. This procedure was repeated iteratively until a final Fe(NH_4_)_2_(SO_4_)_2_ concentration of 8 mM was achieved. Subsequently, the resulting solutions were dialyzed against a storage buffer (50 mM Tris, 500 mM NaCl, pH 8.0) for 48 h to remove precipitated iron.

### 2.6. Electron Microscopy

Samples were prepared on continuous carbon grids (CLC400 Cu25-UT, EM Resolutions Ltd., Keele, ST5 5NP, UK), washed three times with distilled water, and air-dried prior to analysis. For negative staining, grids were treated with 1% uranyl acetate. TEM experiments were performed on a Tecnai T12 Spirit (FEI, now part of Thermo Fisher Scientific, Waltham, MA 02451, USA) at the Faculty of Science Electron Microscopy Facility, University of Sheffield, operated at 100 kV. Carbon contamination in unstained samples was removed using beam showering prior to scanning transmission electron microscopy (STEM) analysis. STEM experiments were carried out on a JEOL ARM200F (JEOL (UK) Ltd., Welwyn Garden City, AL7 1LT, UK) at ePSIC, Diamond Labs, Harwell, operated at 80 kV. For high-resolution STEM (HRSTEM) experiments, a 30 μm probe-forming aperture and spherical aberration correction were used, yielding a semi-convergence angle of 22.6 mrad and a probe size of ~0.1 nm. Additional STEM imaging and spectroscopy was performed on a JEOL F200 (JEOL (UK) Ltd., Welwyn Garden City, AL7 1LT, UK) at the Sorby Centre, University of Sheffield, operated at 200 kV, with a semi-convergence angle of 12 mrad and a collection angle of 17 mrad for bright-field and 53–200 mrad for annular dark-field STEM. Secondary electron (SE) imaging was performed on a Hitachi HF5000 FE-S/TEM (Hitachi High-Tech Europe GmbH, 47807 Krefeld, Germany) at 80 kV, at the Ernst Ruska Centre (ER-C), Jülich, Germany. Additional HRSTEM experiments were conducted on the FEI Titan G2 ChemiSTEM (Thermo Fisher Scientific, Waltham, MA 02451, USA) at ER-C, operated at 200 kV with a convergence angle of 22.4 mrad and collection angles of 69–200 mrad. Finally, 4D-STEM experiments were performed in the same laboratory using a Tescan Tensor (Tescan, 62300 Brno, Czech Republic) at 100 kV.

### 2.7. Data Analysis

#### 2.7.1. Electron Micrograph Analysis

Bright field TEM, HAADF, and SE STEM images were analyzed using *Python3*, enabling efficient batch processing of large numbers of images. Most analyses employed the *scikit-image* [24] and *OpenCV* [25] libraries. For bright-field TEM images, contrast inversion was applied prior to analysis to ensure that strongly scattering regions consistently appeared bright. Pre-processing involved the following three steps: (i) applying a 2 × 2 Gaussian filter to reduce noise, (ii) normalising image contrast to facilitate the generation of binary images of regions of interest (ROIs), and (iii) tiling large images into ROIs to improve computational efficiency. Binary masks of target regions—including protein shells, cores, or iron-nanoparticles—were generated either through global thresholding (Otsu and multi-Otsu) or by first applying a *peak-local-max* function to identify bright features, followed by a *flood-fill* algorithm to delineate boundaries. From these binary images, ROI properties such as particle diameter, area, and mean intensity were extracted for quantitative analysis.

#### 2.7.2. 4D-STEM Data Analysis

4D-STEM spectrum images (SIs) were pre-processed using Otsu thresholding to exclude probe positions outside the encapsulin core regions, thereby removing background and significantly accelerating subsequent data processing. Diffraction patterns at each probe position were plotted on a logarithmic scale to accommodate the large intensity difference between the direct beam and the weaker diffraction spots. The resulting images were subsequently inspected manually to identify diffraction spots.

#### 2.7.3. HRSTEM Data Analysis

For the analysis of HRSTEM images, Digital Micrograph (version 3.6, Gatan, Pleasanton, CA 94588, USA) was used. Images were imported, and the magnification values in the image metadata were calibrated. Lattice planes were identified, and their spacings measured using the line profile tool in Digital Micrograph, integrated over regions 50 pixels wide.

#### 2.7.4. EELS Spectrum Image Analysis

Dual EELS spectra were acquired using JEOL F200 and JEOL ARM200F microscopes. All datasets were processed using Digital Micrograph. The zero-loss peak (ZLP) was identified as the principal peak and aligned at 0 eV. To improve the signal-to-noise ratio, principal component analysis (PCA) was applied to the high-loss (HL) spectra. A ROI was placed over either the nanoparticles or the encapsulin cores, and spectra from all corresponding probe positions were summed to represent the targeted features. The background preceding each ionization edge was modeled using an inverse power-law function preceding the onset of each edge. Elemental quantification was performed using the P L_2,3,_ O K, and Fe L_2,3_ edges, which were analyzed over energy ranges of 132–254 eV, 532–703 eV, and 708–949 eV, respectively. Although the sample thickness was relatively low (*t/λ* < 0.3), plural scattering was removed via low-loss deconvolution using the Fourier-ratio method.

#### 2.7.5. Estimation of the Number of Fe Atoms

The number of Fe atoms was estimated following previously published methods [26,27], using the equation

(1) $$N=\frac{I_{Fe}(\beta ,\Delta )}{I_{0}\left(\beta \right)\;\sigma_{Fe}(\beta ,\Delta )}$$

where *N* is the areal density of atoms (atoms/nm^2^) contributing to the Fe L_2,3_ ionisation edge, *I_Fe_*(β,Δ) is the integral intensity of the Fe L edge, *I*_0_ is the ZLP integral, *β* is the collection angle, ∆ is the signal integration window width, and *σ*_Fe_(*β*, ∆) is the partial inelastic scattering cross-section for Fe L ionisation. The resulting areal density values were subsequently multiplied by the ROI area to obtain the total number of Fe atoms.

#### 2.7.6. Fe L_2,3_ Edge Fingerprinting and Quantification

Reference materials of ilmenite, magnetite, and hematite were used as standards for electron energy-loss near-edge spectroscopy (ELNES). All EEL spectra were collected on a JEOL F200 operated at 200 keV, with a 30 μm condenser aperture, a nominal spot size of 6, and a dwell time of 0.01 s. Dual EELS spectra were acquired with a 660 eV energy offset and a dispersion of 0.1 eV per channel. The resulting spectra were deconvolved using Fourier-ratio deconvolution and background-subtracted using an inverse power law function in the 600–700 eV range. The valence state of iron inside the encapsulin was quantified following the previously established L_3_/L_2_ ratio method [28,29]. Net Fe L_2,3_ intensities were integrated over two 2 eV-wide windows: 708–710 eV for L_3_ and 719.7–721.7 eV for L_2_. A calibration curve of L_3_/(L_3_ + L_2_) versus the L_3_ peak positions was generated using the three reference materials. Experimental data obtained from the encapsulins were then plotted against this calibration curve to estimate the iron valence state.

## 3. Results

### 3.1. Diameters of Native Encapsulins from M. xanthus

**Figure 1:**
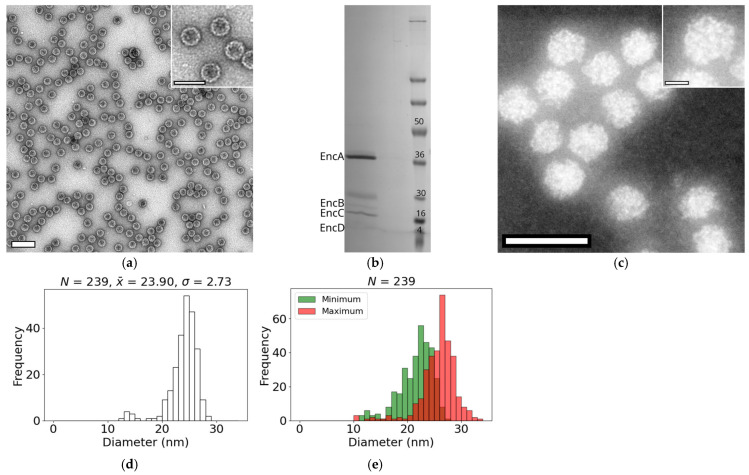
**Isolation and characterization of native encapsulins from *M. xanthus.*** (**a**) Bright-field TEM image of purified native encapsulins, negatively stained with 1% uranyl acetate. Scale bar: 100 nm. The inset shows four encapsulins at higher magnification. Scale bar: 20 nm. (**b**) SDS-PAGE analysis of isolated encapsulins. Bands corresponding to the four encapsulin proteins are labeled: EncA (~32 kDa), EncB (~17 kDa), EncC (~13 kDa), and EncD (~11 kDa). (**c**) HAADF STEM image of encapsulins captured on a JEOL F200 operated at 200 kV. The iron-containing nanoparticles are clearly visualized and conform to the shape of the encapsulin shell. Scale bar: 50 nm. Inset: Single encapsulin highlighting the core region. Scale bar: 10 nm (**d**) Histogram of encapsulin core diameters. (**e**) Histogram of minimum (green) and maximum (red) diameters measured for individual encapsulin cores. For all histograms, the number of particles measured (*n*), the mean ($\bar{x}$), and the standard deviation (*σ*), reported in nm, are shown above the plots.

Native encapsulin nanocompartments were successfully isolated from a yellow-pigmented *M. xanthus* DK1622 wild-type strain. In contrast to previous reports [5], the assembled nanocompartments sedimented as a brown pellet at the bottom of the CsCl gradient rather than forming a whitish band near the top of the CsCl cushion. TEM of negatively stained samples revealed highly homogeneous, spherical particles with an average diameter of ~32 nm (Figure 1a), consistent with earlier observations [5,13]. SDS-PAGE analysis confirmed the nanocompartments are composed of the expected four proteins (Figure 1b): EncA (32 kDa), EncB (17 kDa), EncC (13 kDa) and EncD (11 kDa). Densitometric quantification of the gel bands indicated that each encapsulin shell contains approximately 18 EncB, 61 EncC, and 17 EncD molecules. Compared with previously characterized encapsulins (~36 EncB, ~91 EncC, ~47 EncD [5]), the nanocompartments analyzed here encapsulate fewer cargo proteins overall but retain a high relative abundance of EncC.

Owing to their high atomic number, the iron-rich cores of the encapsulin appear brighter than the surrounding protein shells in HAADF images, facilitating their identification and analysis. Examination of the core regions revealed irregular morphologies, with shapes constrained by the geometry of the surrounding protein shell (Figure 1c). Distinct concavities were observed along the core periphery (Figure 1c: insert), likely corresponding to the positions of the decameric cargo protein assemblies embedded within the encapsulin shell. The average core diameter was 23.9 ± 2.7 nm (*N* = 239), although a small subset of particles exhibited smaller diameters and appeared only partially filled (Figure 1d). Due to the irregular shapes of the cores, we quantified their maximum and minimum diameters, which averaged 26 ± 3 nm (*N* = 239) and 21 ± 3 nm (*N* = 239), respectively (Figure 1e). Notably, atomic models indicate that the encapsulin shell has an internal diameter of approximately 26 nm [5,13,30]. Taken together, these results indicate the entire encapsulin interior can be utilized for iron storage.

### 3.2. Encapsulin Shell Thickness

**Figure 2:**
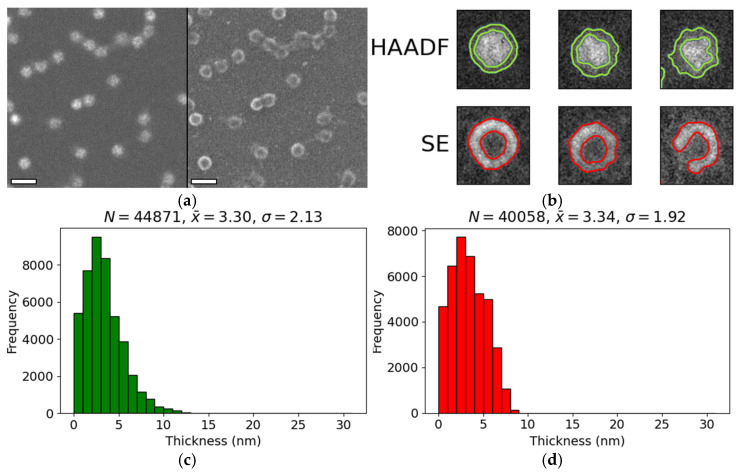
**Measurement of protein shell thickness surrounding encapsulin cores.** (**a**) HAADF and SE images of encapsulins acquired using a Hitachi HF5000 FE-S/TEM at 80 kV. Scale bars: 50 nm. (**b**) Representative individual encapsulins from HAADF or SE images. Outlines corresponding to protein shells identified by image segmentation are shown in green (HAADF) or red (SE). (**c**) Distribution of protein shell thickness measured from HAADF images. (**d**) Distribution of protein shell thickness measured from SE images. Histograms in panels (**c**,**d**) indicate the sample size (*N*), mean ($\bar{x}$), and standard deviation (σ), all in nanometers.

Various imaging modalities, including HAADF and SE STEM, were employed to measure the thickness of the encapsulin shell (Figure 2). In HAADF images, the shells appeared as a medium-intensity band surrounding the bright encapsulin core. In contrast, in SE images the shells were visualised as an elevated ring above a uniform background, although some rings appeared incomplete (Figure 2a). Negative staining revealed that all encapsulins of the sample remained structurally intact, indicating that the apparent discontinuities originated from electrostatic interactions during sample loading. These forces likely caused minor distortion of the protein shell without causing shell breakage, resulting in regions with reduced topographical contrast and therefore lower SE signal.

The average shell thickness measured in HAADF images was 3.3 ± 2.1 nm (*N* = 44,871) (Figure 2c), while SE images yielded a comparable value of 3.4 ± 1.9 nm (*N* = 40,058) (Figure 2d). The close agreement between the two datasets confirms that both imaging modalities interrogate the same structural feature—the encapsulin shell. Furthermore, these measurements are consistent with previously reported structural data [5].

### 3.3. Size and Shape of the Iron-Containing Nanoparticles

**Figure 3:**
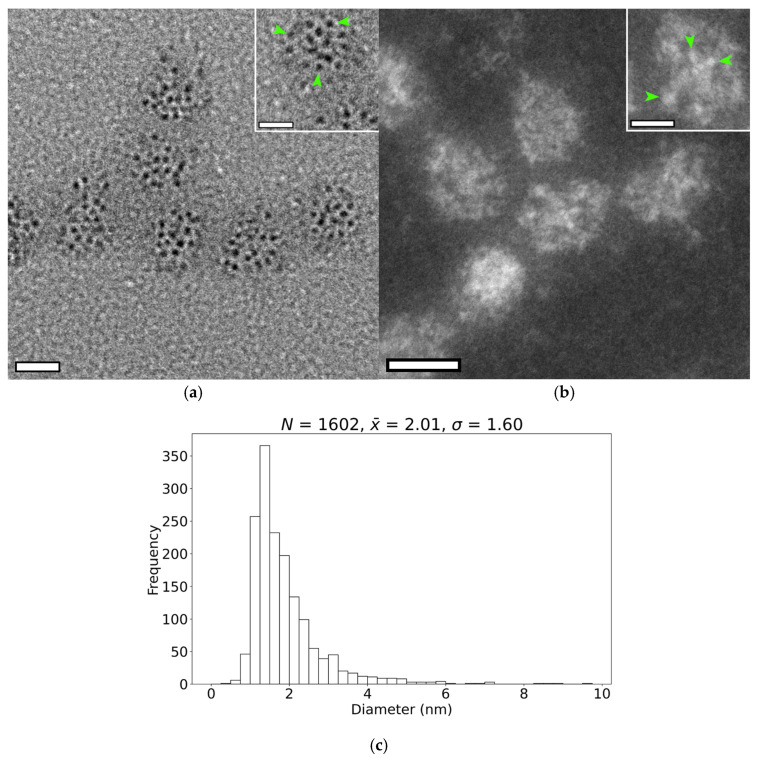
**Imaging and size measurement of the internal nanoparticles.** (**a**) Bright-field TEM image of unstained *M. xanthus* encapsulins. Scale bar: 20 nm Inset: two encapsulins with individual nanoparticles highlighted by green arrows. Image captured with a JEOL F200 at 200 kV. Inset scale bar: 10 nm. (**b**) HAADF STEM image of unstained *M. xanthus encapsulins.* Scale bar: 20 nm. Inset: single encapsulin with individual nanoparticles indicated by green arrows. Image captured on a JEOL ARM200F at 80 kV. Insert scale bar: 10 nm. (**c**) Distribution of nanoparticle diameters measured using both imaging techniques. The sample size (*N*), mean diameter ($\bar{x}$), and standard deviation (*σ*) (all in nm) are reported.

**Figure A1:**
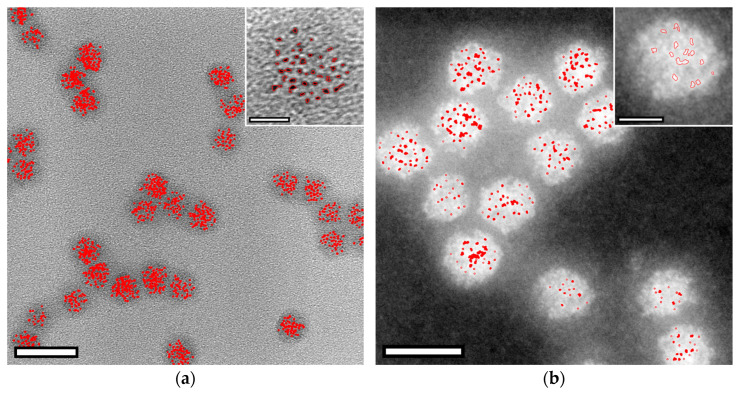
**Identification of nanoparticles from BF TEM and HAADF STEM images.** (**a**) BF TEM image of encapsulins. Scale bar: 100 nm. Insert: a single encapsulin. Scale bar: 10 nm. (**b**) HAADF STEM image of encapsulins. Scale bar: 20 nm. Insert: a single encapsulin. Scale bar: 5 nm. In both images, the image regions defined as nanoparticles after image processing are outlined in red.

Nanoparticles contained within individual encapsulin shells were visualized using both HRTEM (Figure 3a) and HAADF STEM imaging (Figure 3b). In both modalities, the nanoparticles appeared as nearly spherical, high-contrast features clustered within the projected outline of the encapsulin shell in unstained samples. Because nanoparticles can potentially overlap in three dimensions within the encapsulin, simple intensity thresholding was insufficient for identifying the individual nanoparticles. Instead, nanoparticles were detected by identifying the brightest image features, followed by delineation of their boundaries using a flood-fill algorithm (Appendix A.1 Figure A1).

Using this approach, we detected 40 ± 7 (*N* = 13) and 42 ± 13 (*N* = 13) nanoparticles per encapsulin shell in HAADF STEM and HRTEM images, respectively. These values likely represent lower bounds on the true granule count per shell, owing to the difficulty of resolving overlapping granules and the limited depth information inherent to projection imaging. Nevertheless, our results indicate that each shell contains substantially more nanoparticles than the ~15 previously reported [5].

As shown in Figure 3c, the nanoparticles within the *M. xanthus* encapsulin are significantly smaller than previously reported [5], with measured diameters of 2.0 ± 1.6 nm (*N* = 1602). This discrepancy may stem from the enhanced sensitivity of HAADF-STEM imaging for detecting ultrasmall nanoparticles (<3 nm) compared with conventional bright-field TEM [31]. Based on this diameter, the theoretical maximum packing capacity of a single shell is ~2200 nanoparticles, although this estimate neglects the internal volume occupied by cargo proteins and thus exceeds the number that can realistically be accommodated.

We also examined the spatial arrangement of nanoparticles within single shells. No evidence of ordered packing was observed; instead, nanoparticles were distributed without apparent regularity (Figure 3a,b). Nearest-neighbour distance measurements showed that granules were separated, on average, by 1.5 ± 1.3 nm (*N* = 579), suggesting that although no long-range order is present, a minimum spacing between nanoparticles is maintained.

### 3.4. Crystallinity of the Nanoparticles

**Figure 4:**
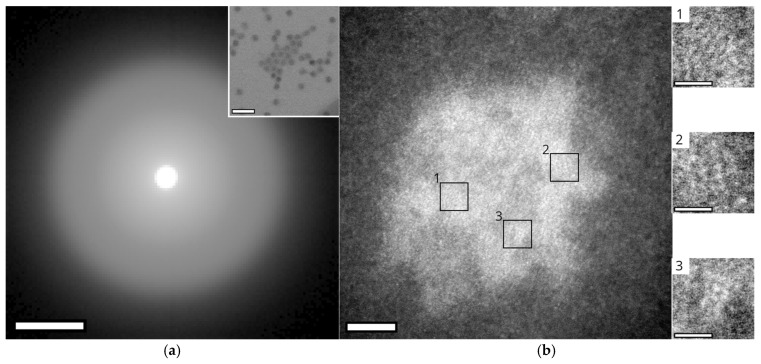
**Crystallinity analysis of nanoparticles within *M. xanthus* encapsulins**. (**a**) 4D-STEM analysis of encapsulated nanoparticles. The summed diffraction pattern of all image pixels corresponding to the encapsulin core shows no discrete diffraction spots; only diffuse rings from the carbon grid are observed, indicating an absence of crystallinity. The image intensity was increased using power-law scaling (contrast range: 0 to 1,280,278, *γ* = 0.3). Scale bar: 0.02 rad. Inset: Reference bright-field image of the analyzed region, showing numerous encapsulins. Images were captured on a Tescan Tensor at 100 kV. Scale bar: 100 nm. (**b**) **Left**: HAADF image of a single encapsulin with three regions of interest showing partially ordered atomic arrangements highlighted. Scale bar: 5 nm. **Right**: Higher magnification images of ROIs 1–3 (**top** to **bottom**). Images were captured using a JEOL ARM200F at 80 kV. Inset scale bars: 1 nm.

The crystallinity of the encapsulated core material was examined using four-dimensional (4D) STEM (Figure 4a) and HR STEM (Figure 4b). For the majority of probe positions analyzed in the 4D-STEM experiments, no diffraction spots were detected; the resulting scattering patterns displayed only diffuse rings consistent with the amorphous carbon support film (Figure 4a). Occasionally, faint partial diffraction spots were observed, but these occurred at only 3 out of ~40,000 probe positions. Taken together, these results indicate that the nanoparticles contained within the encapsulin lack long-range crystalline order.

HRSTEM images of the nanoparticles revealed periodic short-range atomic order in selected regions. Because nanoparticles are superimposed within the encapsulin shell, distinguishing atomic columns belonging to the granules in the upper layer from those beneath is challenging. Nevertheless, several areas exhibiting regular structural features were identified (Figure 4b). These regions contained only a few lattice fringes, typically 3–7, and were no larger than 1.5 nm. The measured interplanar spacings were 160 ± 8 pm, 195 ± 9 pm, 215 ± 11 pm, and 242 ± 12 pm (Appendix A.3). No single iron oxide phase (Appendix A.2 Table A1) can account for this set of spacing; rather, they are consistent with multiple phases, including ferrihydrite (Fe_2_O_3_·0.5H_2_O), hematite (α-Fe_2_O_3_), iron(III) phosphate (α-FePO_4_), and magnetite (Fe_3_O_4_). This multiphasic character resembles that reported for iron oxide cores in isolated ferritin structures [32,33,34].

The partial diffraction spots observed in the 4D-STEM experiments most likely correspond to the sub-nanometre crystalline domains identified by HRSTEM. Owing to the ~1.2 nm spot size limitation of the Tescan Tensor instrument used for 4D-STEM, these small domains were sampled sub-optimally, resulting in poorly resolved diffraction spots at the recorded probe positions.

### 3.5. Chemical Composition of Encapsulin-Derived Materials

**Figure 5:**
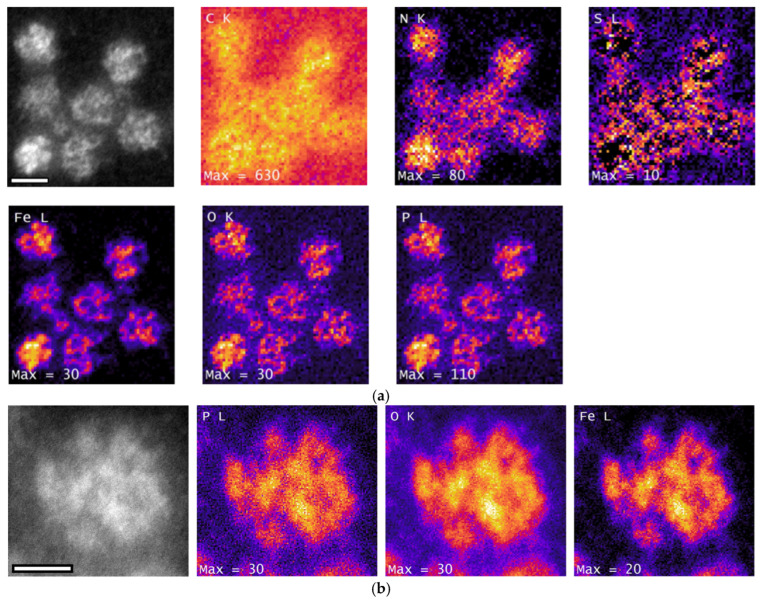

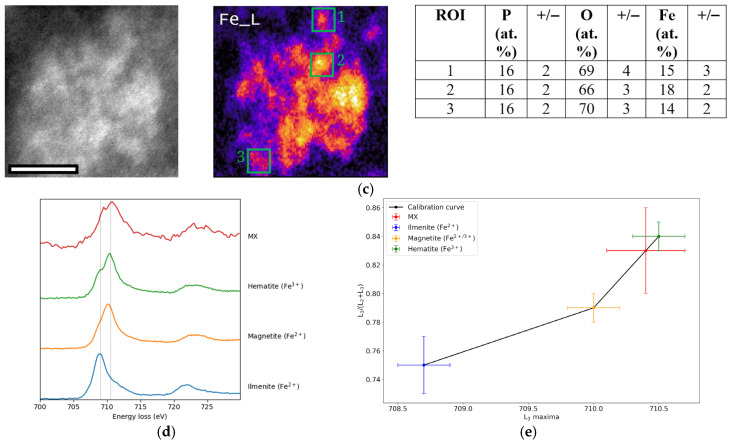
**Chemical composition analysis of nanoparticles within encapsulins.** (**a**) EELS elemental maps of encapsulins. **Top** row, (**left** to **right**): HAADF reference image of the analyzed regions (scale bar: 20 nm), followed by elemental maps for the C K edge, N K edge, and S L edge. **Bottom** row (**left** to *right*): EELS signal intensity maps for Fe L edge, O K edge, and P L edge. Maximum single-pixel intensity values are indicated for each map. (**b**) EELS elemental mapping of individual encapsulins showing signal distribution for the HAADF reference image (scale bar: 10 nm), Fe L edge, O K edge, and P L edge. (**c**) Fe L_2,3_ EELS edge mapping of individual encapsulins. Measurements of individual nanoparticles within the same shell are indicated by green boxes and labelled 1–3. Scale bar: 10 nm. All maps in (**a**–**c**) were captured using a JEOL ARM200F at 80 kV, with ionisation edge signal intensity represented on a temperature-style color scale (black: low intensity; purple to orange: high intensity. (**d**) Fe L_2,3_ edge fingerprinting against reference standards. Vertical dotted lines indicate L_3_ maxima for two end-member reference materials: ilmenite (708.7 eV) and hematite (710.5 eV). “MX” denotes experimental spectra from encapsulins isolated from *M. xanthus.* (**e**) Plot of L_3_/(L_3_ + L_2_) ratio versus L_3_ maxima position. The reference material calibration curve is shown in black, with data points for ilmenite (blue), magnetite (orange), and hematite (green). Experimental data from *M. xanthus* (MX) encapsulins are shown in red. Spectra in (**d**,**e**) were collected using a JEOL F200 at 200 kV.

Analysis of isolated encapsulin cores revealed high enrichment in iron, oxygen, and phosphorus (Figure 5b). Due to the high sensitivity of EELS to light elements and the low electron dose used during acquisition (average: 5.14 ē/Å^2^), the protein shell remained structurally intact throughout data collection. The shell appears as a diffuse, ring-like structure surrounding the iron-dense core in the EELS elemental maps (Figure 5a).

**Figure A2:**
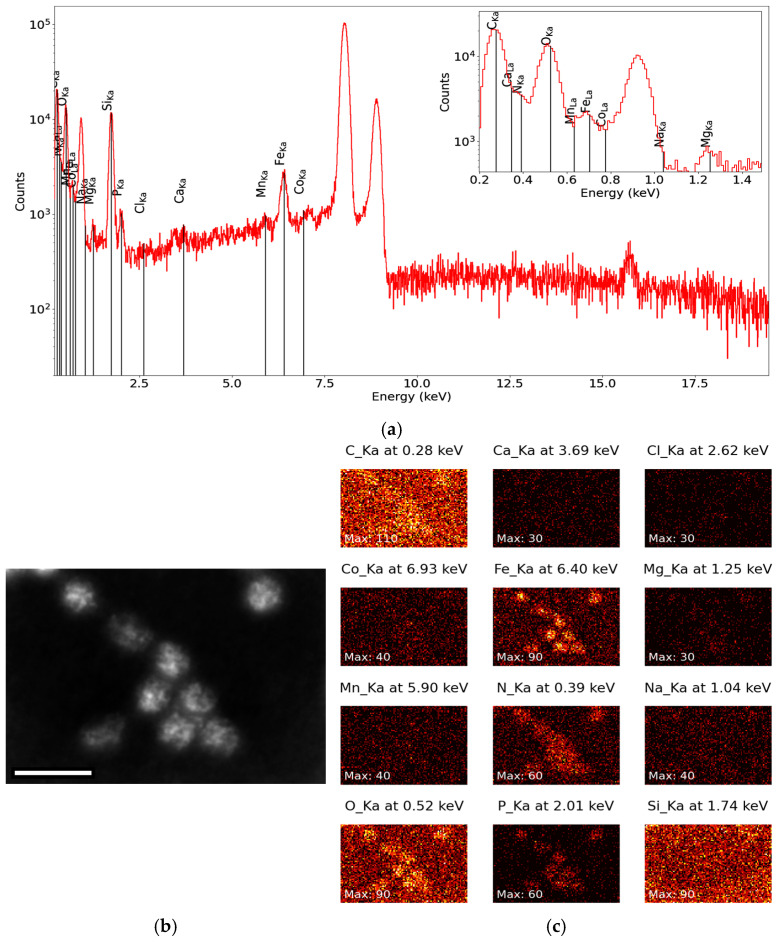
**EDXS investigation of encapsulins.** (**a**) Summed EDX spectra of all probe positions, plotted on a log scale. The peaks representing K lines of all elements are labelled. Insert: 0–1.5 keV. (**b**) HAADF reference image of the investigated region. Scale bar: 50 nm. (**c**) X-ray maps showing the spatial distribution of C_Kα, N_Kα, O_Kα, Na_Kα, Mg_Kα, Si_Kα, P_Kβ, Cl_Kα, Ca_Kα, Mn_Kα, Fe_Kα and Co_Kα. Captured on a Tescan Tensor, operating at 100 kV. The probe size was nominally 0.2 nm with a probe current of 0.53 nA. EDXS maps were collected with a dwell time of 304 ms/pixel over 21 frames, resulting in a total acquisition time of 372 s and a detector dead time of 0.063%. The original maps were binned 10× for display.

EDXS analysis (Appendix A.4 Figure A2) revealed small amounts of Na, Mg, Cl, Ca, Mn, and Co, elements that are typically present at low concentrations in the bacterial cytoplasm. The Mg signal likely originates from MgSO_4_, a major component of the *M. xanthus* CTT growth medium. A weak Cu signal was also detected in association with the encapsulin; however, the limited spatial resolution precludes a definitive assignment of Cu to either the protein shell or the encapsulin core. Notably, this Cu signal persisted even when Cu support grids were replaced with Au grids, suggesting that its presence may be explained by the Irwin-Williams series, which predicts a higher affinity of Cu than Fe for protein ligands [35].

Quantitative EELS analysis of encapsulin cores indicated that the enclosed nanoparticles have a composition consistent with FePO_4_ (Table 1), with an Fe:P ratio of (1.2 ± 0.2):1 (*N* = 21). Higher-resolution imaging of individual encapsulins allowed EELS measurements on multiple individual nanoparticles within single encapsulin shells (Figure 5c), all of which exhibited the same FePO_4_ composition.

Investigation of the Fe L_2,3_ edge using spectral fingerprinting and L_3_/L_2_ ratio analysis was used to determine the iron valence state within the encapsulin. The results indicate that the core material is predominantly Fe^3+^ (Figure 5d,e), although a minor f Fe^2+^ component may also be present. The observed variation in valence state likely reflects, at least in part, electron beam-induced reduction of Fe atoms during imaging, despite the use of low electron doses during imaging [36].

### 3.6. Iron Content

**Figure 6:**
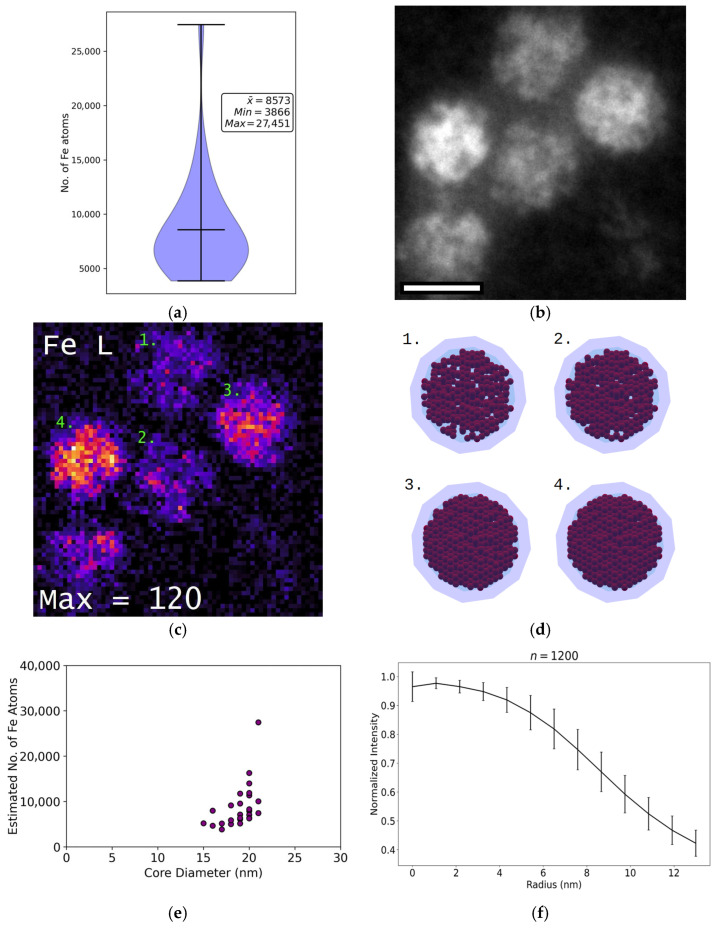
**Iron content of *M. xanthus encapsulins***. (**a**) Violin plot showing the number of Fe atoms detected in encapsulins isolated from *M. xanthus*. (**b**) HAADF reference image of five isolated encapsulins. Scale bar: 20 nm. Image captured using a JEOL ARM200F at 80 kV. (**c**) Chemical EELS map of the same five encapsulins, calculated using the Fe L-edge. Maximum counts per pixel are indicated. Encapsulins are labeled 1–4, corresponding to different Fe loads: 1. 4000 Fe atoms; 2. 8000 Fe atoms; 3. 12,000 Fe atoms; 4. 16,000 Fe atoms. (**d**) Structural models of the four labelled encapsulins in (**c**), illustrating the estimated number of 2 nm Fe nanoparticles derived from Fe atom density (nm^3^) and total Fe content: 1. 444 nanoparticles; 2. 571 nanoparticles; 3. 867 nanoparticles; 4. 941 nanoparticles. The models are simplified and do not include the cargo proteins EncB, EncC, or EncD. (**e**) Correlation plot of average Fe atom number versus measured core diameter. (**f**) Radial integration of encapsulin cores, indicating heterogeneous iron distribution, with the highest concentration at the core centre.

The absolute number of Fe atoms per encapsulin was quantified using EELS. Encapsulins were found to contain between 4000 and 27,500 iron atoms, with an average of 8500 ± 1000 (*N* = 21) iron atoms per shell (Figure 6). Volumetric iron density varied across different encapsulin shells, ranging from 1.4 to 5.5 atoms/nm^3^, with a mean density of 2.1 ± 0.7 atoms/nm^3^ (*N* = 21). In contrast, no significant variation was observed between nanoparticles within the same encapsulin shell. Based on the highest measured density, the maximum number of iron atoms that could be stored in a single *M. xanthus* encapsulin is estimated at ~50,000 iron atoms, assuming inter-nanoparticle spacing is not maintained.

Consistent with expectations, encapsulins with higher iron content exhibit brighter cores in HAADF images (Figure 6b,c). However, core size and Fe atom number were poorly correlated (*R*^2^ = 0.2) (Figure 6b), with measured core diameters ranging from 15–21 nm across the observed iron content range. This indicates that factors beyond total iron load influence the spatial distribution of nanoparticles within the encapsulin. Radial integration of HAADF images (Figure 6f) revealed an uneven distribution of iron within the encapsulin cores, with the highest density of iron atoms located at the centre.

### 3.7. Phosphorus Content

**Figure 7:**
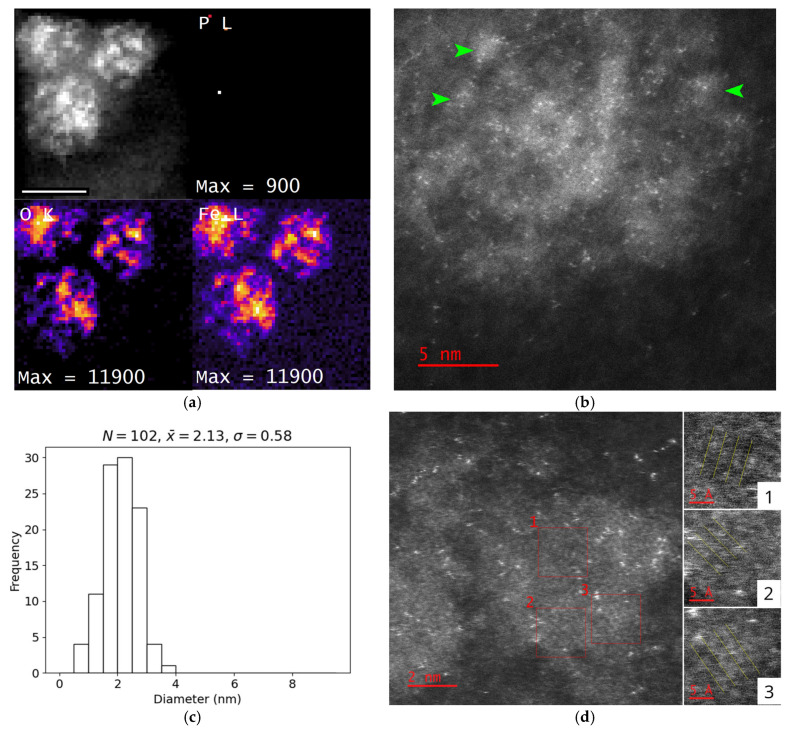
**Role of phosphorus within the encapsulin** (**a**) Chemical maps of encapsulins generated from an EEL spectrum image. The absence of phosphorus in the core is indicated by the lack of signal in the P L-edge map. Spectrum images were collected using a JEOL F200 at 200 kV. Scale bar: 20 nm. (**b**) HAADF reference image of a single encapsulin obtained using an FEI ChemiSTEM at 80 kV. Individual nanoparticles within the encapsulin are indicated by green arrows. (**c**) Histogram showing the average distribution of measured nanoparticle diameters. The number of particles measured (*n*), the mean diameter ($\underline{x}$), and the standard deviation (σ) are provided (all in nm). (**d**) **Left**: HAADF HRSTEM image of encapsulins captured using an FEI ChemiSTEM at 80 kV. The three ROIs exhibit a periodic atomic arrangement. *Right*: higher-magnification images of the three ROIs (top to bottom: ROIs 1–3), with lattice planes are highlighted by yellow lines. Scale bars: 5 Å.

**Figure A3:**
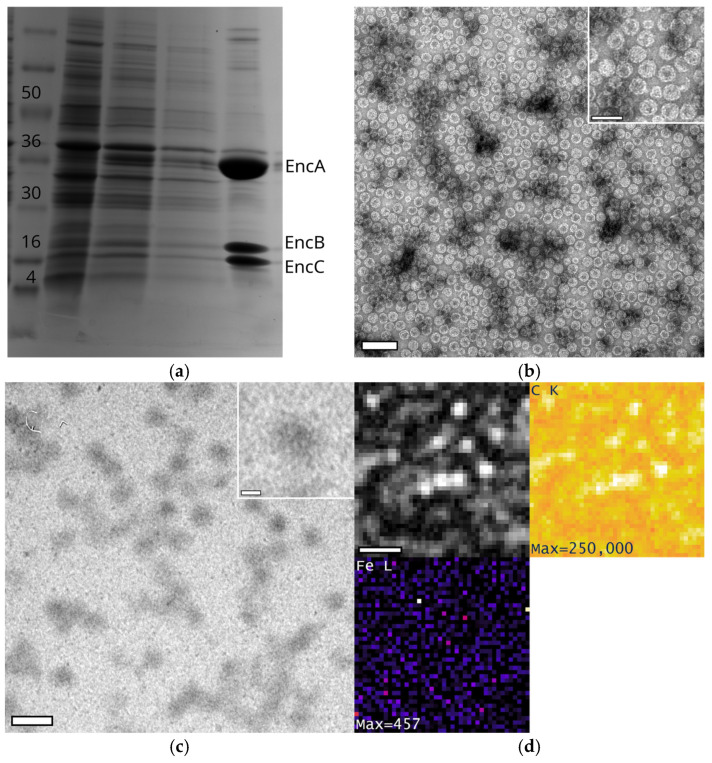
Generation and isolation of EncA + EncB + EncC construct from *E. coli.* (**a**) SDS-PAGE analysis of encapsulin expressed in *E. coli*. Lanes are labelled 1–5 from left to right. Lane 1: See Blue Prestained protein ladder (Invitrogen). Lane 2: Cell lysate. Lane 3: first wash. Lane 4: second wash. Lane 5: EncA (36 kDa), EncB (17 kDa) and EncC (13 kDa). (**b**) Electron micrographs of generated encapsulin construct, negatively stained with 1% uranyl acetate. Scale bar: 100 nm. Inset: a selection of negatively stained encapsulins. Scale bar: 50 nm. Images were captured on a Technai Spirit at 100 kV. (**c**) Unstained electron micrograph of EncA + EncB + EncC before iron loading. The lack of observable electron dense cores inside the low contrast shell is evidence that minimal iron loading occurs during expression inside *E. coli*. Scale bar: 50 nm. Inset: a representative example of a single empty shell. Scale bar: 10 nm. Images were captured on a Technai Spirit at 100 kV. (**d**) EELS signal maps of EncA + EncB + EncC encapsulins before iron loading. The images are an HAADF reference image (top left), the carbon K-edge map (top right) and the iron L-edge map (lower left). The presence of only background noise in the Fe L-edge map indicates that there is minimal iron loading. Spectrum image captured with a JEOL F200 operating at 200 kV.

To assess whether phosphorus in the encapsulin core influences the crystallinity of the stored material, empty encapsulins containing both EncB and EncC were loaded with 8 mM of Fe(NH_4_)_2_(SO_4_)_2_ under phosphorus-free conditions (Appendix A.5, Figure A3). Prior to iron loading, isolated EncA + EncB + EncC encapsulins were confirmed to lack iron-rich cores using unstained BF-TEM imaging and EELS mapping (Appendix A.5, Figure A3). Introduction of iron under these conditions resulted in the accumulation of nanoparticles with an average diameter of 2.1 ± 0.6 nm (*N* = 102) within the encapsulins (Figure 7b,c). EELS chemical mapping confirmed the absence of phosphorus in the resulting core material (Figure 7a). HR-STEM HAADF imaging revealed that these nanoparticles exhibited crystalline features, with the largest continuous crystalline domain measuring 2.6 nm (Figure 7d). Measurements of nanoparticles within ten encapsulin shells indicated multiple inter-planar spacings: 176 ± 8 pm, 204 ± 8 pm, 224 ± 11 pm, 278 ± 13 pm, and 300 ± 15 pm. These spacings are consistent with a single iron oxide phase, goethite (α-FeO(OH)) (Table A2). Elemental quantification further supports the presence of goethite, showing an average composition of 36 ± 3% Fe (*N* = 28) and 64 ± 3% O (*N* = 28), with hydrogen undetectable. Due to the complex arrangement of the nanoparticles within the encapsulin shells, it was not possible to determine whether individual nanoparticles were mono-crystalline or, more likely, poly-crystalline.

**Figure A4:**
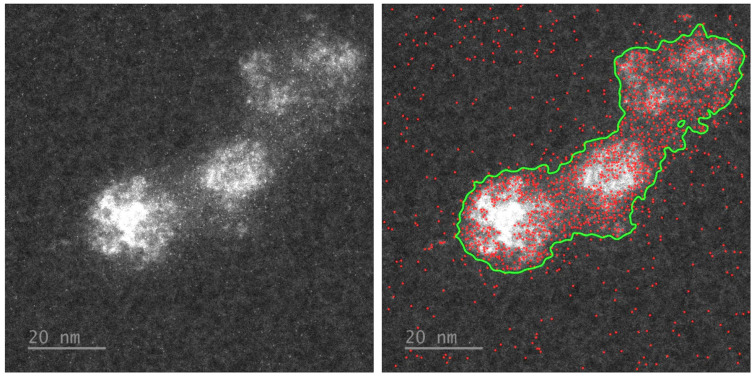
**Distribution of bright spots in HAADF images of EncA + EncB + EncC encapsulins loaded with iron in a phosphorus-free environment. Left:** HAADF reference image of encapsulins **Right:** Same image with overlay showing the distribution of bright nanoparticles (red). The boundary of the encapsulin protein is outlined in green. Scale bars: 20 nm.

Across the collected HRSTEM images, discrete bright spots are observed (Figure 7b). While these features are distributed throughout the sample, their density is significantly higher in the vicinity of the encapsulin protein shell, with 75% of bright spots within the investigated region localised to the encapsulin protein (Appendix A.6 Figure A4). The bright spots exhibit an average size of 0.20 ± 0.07 nm (*N* = 213) and an average brightness roughly 16.5 times that of a single carbon atom, determined from a measured carbon film thickness of 3.8 nm for t/λ = 0.06 and an atomic density of 36 atoms/nm^3^. For the large inner collection used, the HAADF image intensity is estimated to scale with the atomic number, *Z*, to the power of ≈1.9, indicative of nearly pure Rutherford scattering [37]. Based on these results, the bright spots are consistent with single iron atoms or ions [38]. Their preferential localization to protein-rich regions suggests binding to the encapsulin protein surface; however, no discernible spatial pattern was identified.

## 4. Discussion

The results presented here offer new insights into the iron-containing mineral stored inside the encapsulin of *M. xanthus.*

### 4.1. Encapsulin Content

The isolated encapsulins contained fewer cargo proteins than previously reported in either native structures [5] or from heterologously expressed systems [13]. In both of these cases, the cargo proteins accounted for ~30% of the protein total content of the encapsulin system and it was confirmed that the cargo protein binding sites were not fully occupied. The reduced number of cargo proteins in the structures isolated here suggests that there is more unoccupied space inside the encapsulin shell, potentially allowing for increased iron storage. Consistent with this, the encapsulins isolated in the present study contained a larger number of nanoparticles than previously reported and were found to pellet at the bottom of the CsCl gradient during isolation, rather than forming a white band in the gradient [5]. These results indicate that the encapsulins isolated here were more heavily loaded with iron. This may result from the specific experimental conditions used to induce starvation in *M. xanthus* cultures and suggests the presence of a previously uncharacterized mechanism regulating iron loading into the encapsulin shell, beyond the currently accepted ‘passive diffusion’ model [4,5,39].

### 4.2. HAADF and SE Measurements of the Protein Shell

The dimensions of the encapsulin protein shell were determined using a novel combination of HAADF and SE imaging. Our measurements are consistent with previous data derived from the atomic model of the encapsulin shell [5] and confirm that the entire lumen of the encapsulin is available for nanoparticles storage. Although these techniques are not routinely used due to the high beam sensitivity of biological materials under STEM [40,41], both proved effective for measuring protein dimensions, even in the absence of high spatial resolution. Especially SE imaging allowed clear identification of the inner edge of the encapsulin protein shell, facilitating accurate measurements of shell thickness and providing a useful technique for the structural characterization of encapsulins.

### 4.3. Nanoparticle Morphology

The encapsulins investigated in this study contained 2 nm small spherical nanoparticles that were generally amorphous in their atomic structure. These nanoparticles, when compared to the previously observed 5 nm nanoparticles [5] with a proposed crystalline arrangement [13], are amorphous and smaller, which offers distinct functional advantages in the context of oxidative stress response. Smaller nanoparticles possess a higher surface area to volume ratio, enhancing their chemical reactivity, and iron from amorphous compounds is more readily mobilised than from crystalline forms [42,43]. Consequently, the physical properties of these encapsulated nanoparticles likely facilitate rapid iron accumulation and release, enabling cells to respond efficiently to environmental changes—either mitigating oxidative stress under nutrient-limited conditions or resuming vegetative growth upon the restoration of nutrient availability.

HR-STEM analysis of the encapsulin containing EncB and EncC loaded with iron in a phosphorus-free environment showed small regions with nano-crystalline arrangements and the measured d-spacings of the mineral goethite. Previous work has identified that the cores of encapsulins isolated from bacteria contain phosphorus [5,6], and phosphorus is known to affect the crystallization of the iron oxide nanoparticles inside ferritin cages [44,45]. Thus, the presence of phosphorus is implicated in the maintenance of non-crystalline material inside the encapsulin and therefore the presence of more easily mobilized iron within the structure, similar to other ferritin systems.

### 4.4. Nanoparticle Chemistry

Additionally, it has been identified that the material inside the encapsulin has a general formula of Fe^3+^PO_4_, plus potentially an unknown amount of hydrogen which cannot be detected. Thus, the material is an oxy-hydroxide-phosphate similar to the material that has been observed inside other ferritin systems [46,47,48,49]. The average Fe:P ratio in the nanoparticles was calculated as 1.2:1, closely matching that of *Q. thermotolerans* encapsulin, where a Fe:P ratio of 1:1 was measured using STEM-EELS [6], and is also more similar to measurements made from bacterioferritin systems where a ratio of 1–2:1 is often observed [50,51,52]. In contrast, it differs markedly from previous measurements in *M. xanthus*, where a Fe:P ratio of 4:1 was recorded using inductively coupled plasma mass spectrometry [5]. Due to the dynamic nature of intracellular phosphorus levels and the unknown P content of *M. xanthus* cell, this difference is likely due to differences in the cellular environment during encapsulin expression. Spatially resolved STEM-EELS experiments indicate that this general formula is consistent at the individual nanoparticle level, suggesting that all nanoparticles within a single encapsulin share the same chemical composition.

Encapsulins were found to contain on average, about 8500 Fe atoms, substantially less than the previously reported maximum of 30,000 atoms [5] or the maximum of 50,000 atoms estimated in the present study. This discrepancy may be attributable to differences in the broader cellular context at the time of expression. Given that encapsulin expression is upregulated during starvation, it is plausible that these proteins are synthesized rapidly by the cell in response to environmental stress, resulting in accelerated Fe uptake. Additionally, a lower Fe loading density could promote more rapid Fe release once normal growth conditions are restored, enabling the cell to adapt rapidly to fluctuations in environmental nutrient availability.

## 5. Conclusions

In conclusion, through both electron microscopic and spectroscopic analysis, we determined that the internal nanoparticles stored inside the *M. xanthus* encapsulin are much smaller than previously identified (2 nm), more numerous (theoretical maximum of 2200 nanoparticles per shell) and generally amorphous with a general formula FePO_4_. It was also identified that the structures contain on average 8500 iron atoms and that phosphorus plays a key role in preventing the formation of crystalline material inside the encapsulin. These findings suggest that the *M. xanthus* cell can utilize encapsulins for iron storage in response to oxidative stress and in performing general iron homeostasis much more rapidly than previously understood. Possessing nanoparticles that are smaller than previously identified and structurally amorphous instead of crystalline, means that iron can be more rapidly stored and liberated from these structures than previously understood, which has important implications for our understanding of how encapsulins are implicated in responding to oxidative stress. More broadly, this work advances our understanding of the response of the *M. xanthus* cell to decreases in nutrient levels in its environment.

## Figures and Tables

**Table 1:** Elemental composition of *M. xanthus* encapsulin nanoparticles. The values were calculated via absolute quantification of EELS data.

Element	at. %	+/− (Std. Dev.)
Fe	15	2
O	75	4
P	12	3

## Data Availability

The raw data supporting the conclusions of this article will be made available by the authors on reasonable request.

## Abbreviations

The following abbreviations are used in this manuscript:

BF	bright field
CTT	casitone tris growth medium
dd	double distilled
EDXS	energy-dispersive X-ray spectroscopy
EELS	electron energy-loss spectroscopy
HAADF	high angle annular dark field
HR	high resolution
M.	Myxococcus
RPM	revolutions per minute
SE	secondary electron
TEM	transmission electron microscopy
TPM	tris-phosphate magnesium starvation medium
STEM	scanning transmission electron microscopy

## Appendix A

### Appendix A.2. Published Iron Oxide Lattice Parameters

**Table A1:** Iron oxide lattice parameters.

Valence	Chemistry	Name	Space Group	No.	Comment	Lattice Parameters (pm)	Source
II	FeO	wuestite	Fm3m	225	cubic	*a* = 430	[53]
II/III	Fe_3_O_4_	magnetite	Fd3m	227	cubic, inverse spinel	*a =* 840	[54]
III	α-Fe_2_O_3_	hematite	R3c	167	hexagonal	*a =* 504, *c* = 1375	[55]
	γ-Fe_2_O_3_	maghemite	Fd3m	227	cubic, spinel	*a* = 833	[56]
	α-FeO(OH)	goethite	Pbnm	62	orthorhombic	*a* = 460, *b* = 995, *c* = 302	[57]
	Fe_2_O_3_-0.5H_2_O	6-line ferrihydrite	P63mc	189	hexagonal	*a =* 296, *c* = 940	[58]
	α-FePO_4_	iron (III) phosphate	P3_1_21	152	trigonal	*a* = 500, *c* = 1125	[59]

### Appendix A.3. Measured Lattice Fringes and Phase Assignments

**Table A2:** Table of the measured *d*-spacings from HR-STEM images of the nanoparticles inside encapsulins isolated from *M. xanthus.* The errors represent the uncertainty when measurements were made from selected line profiles. Different iron oxides phases are colour-coded: ferrihydrite (red), goethite (blue), hematite (green), iron(III) phosphate (purple), magnetite (orange), maghemite (brown) and wuestite (black).

*d*-Spacing (pm)	Error (pm)	Matching Phases and Miller Indices hkl (in Brackets)	Predicted *d*-Spacing (pm)
		ferrihydrite (123)	163
		goethite (300)	153
		hematite (123)	155
		hematite (210)	165
		hematite (312)	160
		iron (III) phosphate (311)	160
		iron (III) phosphate (122)	155
		magnetite (333)	162
		wuestite (022)	152
195	9	ferrihydrite (013)	198
		hematite (214)	203
		hematite (223)	197
		hematite (114)	203
		iron (III) phosphate (022)	199
		magnetite (024)	188
		magnetite (133)	193
		maghemite (024)	186
		maghemite (133)	191
215	11	ferrihydrite (112)	225
		goethite (220)	209
		goethite (210)	224
		hematite (-202)	208
		iron (III) phosphate(112)	225
		iron (III) phosphate (200)	214
		iron (III) phosphate (201)	210
		iron (III) phosphate (113)	205
		magnetite (004)	210
		maghemite (004)	208
		wuestite (002)	215
242	12	ferrihydrite (011)	248
		ferrihydrite (004)	248
		goethite (200)	230
		goethite (101)	252
		goethite (111)	245
		hematite (112)	236
		hematite (211)	247
		hematite (111)	247
		hematite (122)	236
		iron (III) phosphate (110)	247
		iron (III) phosphate (111)	241
		iron (III) phosphate (014)	232
		magnetite (222)	242
		magnetite (113)	253
		maghemite (222)	240
		maghemite (113)	251
		wuestite (111)	248
